# Impact of umbilical fixation on perfusion in abdominoplasty: A hyperspectral imaging study

**DOI:** 10.1016/j.jpra.2025.06.015

**Published:** 2025-06-30

**Authors:** Moritz Künzel, Lukas Kohler, Hannes Köhler, Torsten Schulz, Rima Nuwayhid, Georg Osterhoff

**Affiliations:** a Department of Orthopaedics, Trauma and Plastic Surgery, University Hospital Leipzig, Leipzig, Germany; b Innovation Center Computer Assisted Surgery (ICCAS), University of Leipzig, Leipzig, Germany

**Keywords:** Abdominoplasty, Umbilicus, Wound healing, Hyperspectral imaging, Tissue perfusion

## Abstract

**Background:**

Postoperative necrosis of the umbilicus is a rare yet serious complication in abdominoplasty. Fixation of the umbilicus to the fascia (umbilicopexy) is commonly used to enhance aesthetic outcomes, but concerns regarding potential compromise to perfusion remain unaddressed. This study investigates the impact of umbilicopexy on umbilical perfusion using hyperspectral imaging (HSI), a non-invasive method for assessing tissue oxygenation and perfusion.

**Methods:**

In this prospective study, 28 patients (25 female; mean age 42 years, range 24 to 60 years) undergoing abdominoplasty were assessed: 11 underwent umbilicopexy, while 17 served as controls. HSI parameters, including tissue oxygenation (StO_2_), near-infrared perfusion index (NIR PI), tissue hemoglobin index (THI), and tissue water index (TWI), were recorded intraoperatively and on postoperative days (POD) 1 and 2. Clinical outcomes, including marginal wound necrosis and revision surgeries, were also documented.

**Results:**

StO_2_ values were higher intraoperatively in the umbilicopexy group (80 ± 7 %) compared to the control group (68 ± 16 %; *p* = 0.025). By POD 1, StO_2_ decreased in both groups (umbilicopexy: 66 ± 11 %, control: 56 ± 15 %, *p* = 0.067). Marginal wound necrosis occurred in 9 % of the umbilicopexy group and 18 % of controls (*p* = 1.0), while revision surgery related to perfusion deficits was required in none of the patients in both groups. No significant differences were found in NIR PI, THI, or TWI across groups or time points.

**Conclusion:**

HSI proved valuable for real-time perfusion monitoring suggesting that Umbilicopexy is not adversely affecting umbilical perfusion or wound healing in abdominoplasty. Larger cohort studies with additional confounders are recommended to confirm these findings.

## Introduction

Postoperative necrosis of the umbilicus describes a rare but serious complication in umbilicoplasty during abdominoplasty, leading to a severe disturbance in wound healing. A plethora of techniques have been described to achieve a stable and aesthetically pleasing result after reinsertion of the umbilicus into the abdominal wall. In 40 % of the described techniques, the dermis of the umbilical skin is sutured to the fascia of the rectus abdominis muscle to achieve an umbilical groove with sufficient depth.^1^ Data on results and complications are lacking. Furthermore, no clear indication for this technique has been defined. Thus, this is usually performed by individual choice of the surgeon based on experience. The consistent achievement of satisfactory results has led to the regular application of this technique is in our clinic. In the absence of an internationally agreed term for this procedure, we internally established the term *umbilicopexy*. Umbilicopexy might affect umbilicus perfusion, leading to complications. The risk for postoperative circulatory disorders of the umbilicus after umbilicoplasty could be elevated in post-bariatric patients due to lengthening of the umbilical stalk. In the early postoperative days, the umbilicus is usually monitored by clinical assessment during the change of dressing to detect, among other complications, impairment of umbilical perfusion. There is no established tool to monitor perfusion. Hyperspectral imaging (HSI) is a well established, non-invasive method to detect changes in the tissue by combining spectroscopy and imaging. It has already proven its diagnostic value in several clinical studies and has established itself in our clinical work where it was used as a monitoring tool to allow early detection of changes in the tissue.^2^^,^^3^ The current study aimed to objectively investigate, whether suturing the umbilicus to the rectus abdominis muscle fascia during abdominoplasty has a negative effect on umbilical perfusion measurable via HSI.

## Patients and methods

### Patients

The protocol of this prospective diagnostic study was approved by the local ethics committee (reference 426/19-ek). Patients aged 18 and older, who underwent abdominoplasty with umbilicoplasty between October 2019 and November 2020 and gave informed consent, were included. In total, 28 consecutive patients (25 female, 3 male) with a mean age of 42 years (SD 11 years, 24 to 60 years) were included (Table 1). Patients who declined participation and those where additional reconstructive interventions around the abdominal wall were performed (*n* = 1), were excluded. Fixation of the umbilicus to the fascia of the abdominal wall was performed in 11 patients (umbilicopexy) as per surgeon’s choice, while the umbilicus was sutured solely to the skin in 17 patients (control). No plication was performed around the umbilicus.

**Table 1:** Baseline characteristics.

	Control	Umbilicopexy	*p*
N	17	11	
Age [y]	41, SD 11	44, SD 11	0.405
Sex [f:m]	16:1	9:2	0.543
Obesity scale			0.440
Overweight (BMI 25.0–29.9)	2	3	
Obesity I (BMI 30.0–34.9)	7	5	
Obesity II (BMI 35.0–39.9)	8	2	
Obesity III (BMI >40.0)	0	1	
Indication			0.167
Weight-loss by lifestyle	6	5	
Weight-loss by bypass surgery	4	5	
Post-pregnancy	7	1	
Hospitalization after surgery (d)	6, SD 2	6, SD 2	0.964
Comorbidities [n]			
Diabetes	0	2	0.146
Arterial hypertension	4	5	0.409
Smoking	3	1	1.0

For umbilicopexy, the skin surrounding the umbilicus is incised in a circular shape. When elevation of the abdominal flap reaches the umbilicus, it is dissected leaving a cuff of adipose tissue around the stalk. The dermis of the umbilical skin flap is then sutured to the rectus abdominis muscle fascia with four cardinal stitches using non-absorbable suture. The umbilical flap’s skin is later sutured to the border of a U-shaped incision in the skin of the abdominal wall. When omitting umbilicopexy, the umbilical flap’s skin is directly sutured to the border of the incision in the abdominal wall.

## Data acquisition

During the surgery after transposition of the umbilicus and its fixation to the fascia in the intervention group as well as on postoperative days 1 and 2, images of the umbilicus with periumbilical area were taken using the hyperspectral imaging system (TIVITA® Tissue, Diaspective Vision GmbH, Germany.)

**Figure 1:**
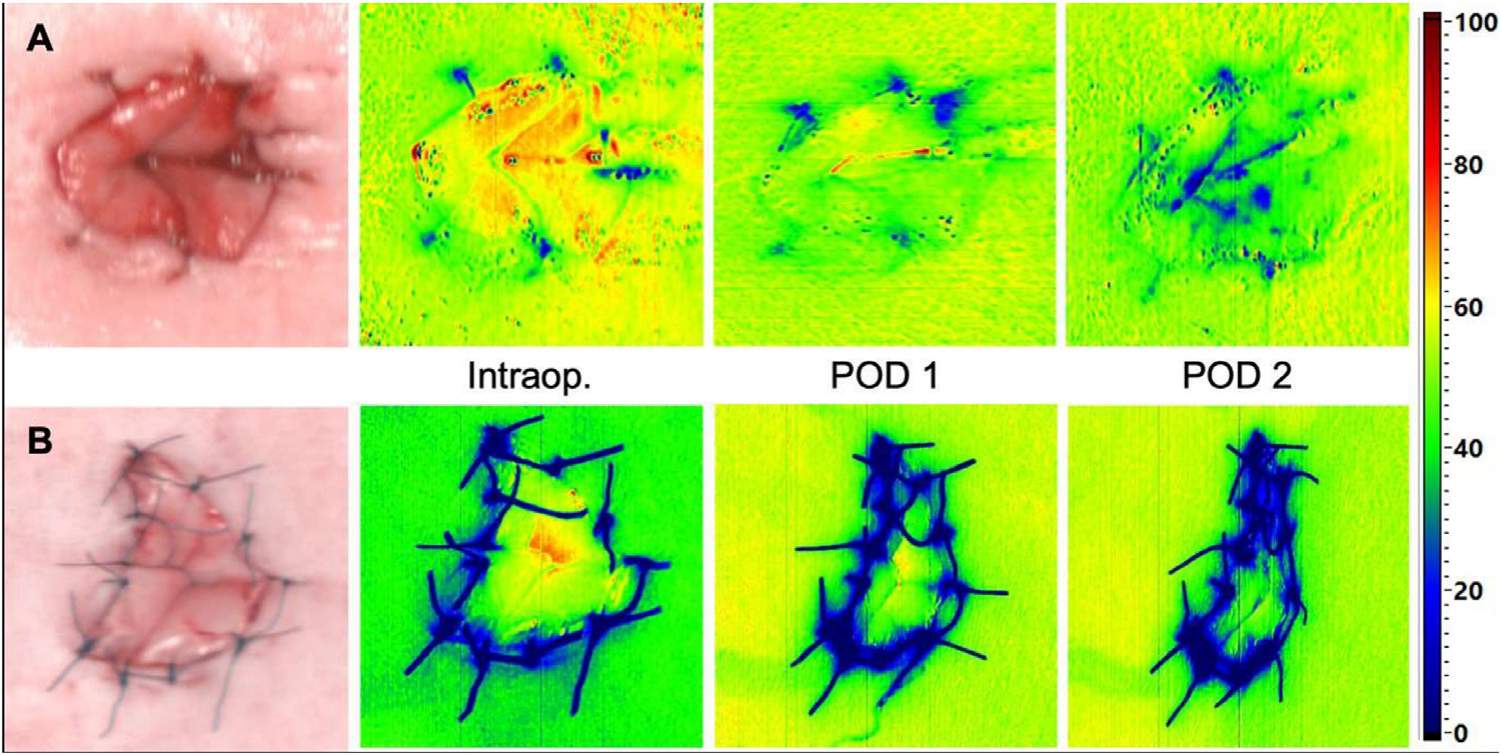
Hyperspectral imaging-acquisition (example of hyperspectral imaging-acquisition (RGB images, NIR PI) of the umbilicus (A) control and (B) with umbilicpexy.

The system employs a built-in halogen light source to acquire reflectance spectra from 500 to 1000 nm in 6.4 s. This data is processed in real-time, producing false-color images that represent physiologic tissue parameters in the range from 0 to 100. These physiological ranges were previously described and evaluated by Holmer et al.^4^ The calculations included tissue oxygenation (StO2) and near-infrared perfusion index (NIR PI, Figure 1), tissue hemoglobin- (THI), and tissue water index (TWI) were calculated. The field of view (FOV) for all parameters was 21 × 30 cm^2^ and the spatial resolution was 0.56 mm at 630 nm, as evaluated using the 1951 USAF resolution test chart at 50 cm object distance).

At all time points an experienced fellowship-trained surgeon assessed the umbilical and defined areas of marginal wound necrosis or–if necessary–made the decision for wound revision.

### Statistical analysis

Prior to recruiting, a sample size calculation was conducted. Assuming a clinical difference between the groups in HSI parameters of 20 and a standard deviation of 15 and aming for a power of 0.90 with an α error of 0.05 it revealed a minimum sample size of 10 per group.

All post-hoc calculations were performed with TIVITA® Suite and SPSS 29.0. Continuous data are presented as mean and standard deviation (SD), nominal data in frequencies and percentages. After histogrammatic testing for normal distribution, either a paired Student’s-t-test or paired non-parametric tests were used to detect differences in means of continuous data. For comparison of categorical data, cross-tables and Pearson-Chi-Square and Fisher’s Exact tests were used. An Analysis of Variance (ANOVA) was performed for the comparison of repeated HSI measurements. The level of significance was defined as *p* < 0.05.

## Results

### Baseline data

Marginal wound necrosis was observed in 4 patients. Surgical wound revision was necessary in 2 patients in the umbilicopexy group, one for hematoma on postoperative day 1 and one for surgical site infection on postoperative day 15. There were no revisions due to wound necrosis or delayed wound healing secondary to poor perfusion (Table 2).

**Table 2:** Clinical outcome parameters.

	Control	Umbilicopexy	*p*
			
N	17	11	
Marginal wound necrosis	3 (18 %)	1 (9 %)	1.0
Revision surgery	0 (0 %)	2 (18 %)	0.146
related to poor perfusion	0 (0 %)	0 (0 %)	1.0

### Hyperspectral imaging

**Figure 2:**
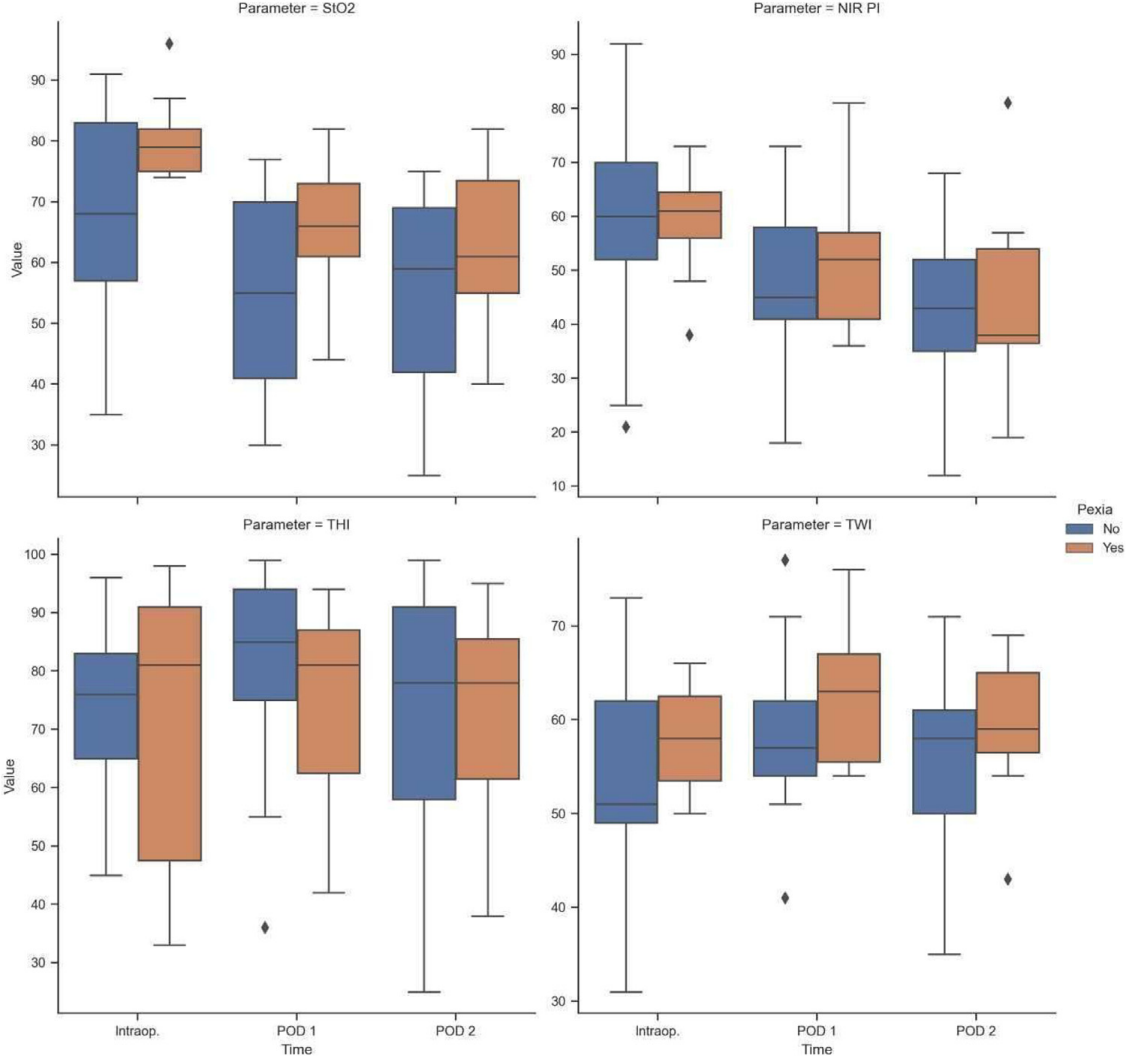
Tissue oxygenation (StO_2_), near-infrared perfusion index (NIR PI), tissue hemoglobin-index (THI) and tissue water-index (TWI) across the two patient cohorts at various observation time points (INTRAOP: intraoperative, POD1: postoperative day 1, POD 2: postoperative day 2, blue: without umbilicopexy, brown: with umbilicopexy).

The NIR PI (*p* < 0.001) and StO_2_ (*p* < 0.001) both decreased over time from intraoperatively to POD 2 (Table 3, Figure 2). No change over time was seen for the THI (*p* = 0.060) and the TWI (*p* = 0.050, Table 3, Figure 2).

**Table 3:** Hyperspectral imaging outcome parameters.

	Control	Umbilicopexy	*p*
N	17	11	
Near-infrared perfusion index (NIR PI)			
Intraoperatively	58, SD 19	59, SD 10	0.857
POD 1	46, SD 16	51, SD 13	0.409
POD 2	43, SD 15	44, SD 17	0.912
Tissue oxygenation index (StO2)			
Intraoperatively	68, SD 16	80, SD 7	0.025
POD 1	56, SD 15	66, SD 11	0.067
POD 2	54, SD 17	63, SD 13	0.149
Tissue hemoglobin index (THI)			
Intraoperatively	74, SD 14	72, SD 25	0.746
POD 1	81, SD 17	74, SD 18	0.302
POD 2	71, SD 24	73, SD 20	0.894
Tissue water index (TWI)			
Intraoperatively	55, SD 11	58, SD 6	0.442
POD 1	58, SD 8	62, SD 8	0.220
POD 2	56, SD 9	60, SD 7	0.246

The intraoperative StO_2_ was higher in the umbilicopexy group compared to the control (80, SD 7 vs 68, SD 68, *p* = 0.025) (Table 1, Figure 2). For all other time points and all other HSI parameters (NIR PI, THI, TWI) there were no significant differences between umbilicopexy group and control (Table 2, Table 3, Figure 2).

## Discussion

This study evaluated the potential impact of umbilical fixation to the rectus abdominis fascia (umbilicopexy) on umbilical perfusion following abdominoplasty using hyperspectral imaging (HSI). Our findings suggest that umbilicopexy does not adversely affect umbilical perfusion, as evidenced by comparable tissue oxygenation indices and perfusion metrics between the intervention and control groups.

The absence of significant differences in clinical outcomes, such as marginal wound necrosis and the need for revision surgery due to perfusion deficits, further suggests the safety of umbilicopexy. Although two revision surgeries were reported in the umbilicopexy group, these were unrelated to umbilical perfusion deficiencies. HSI measurements, specifically tissue oxygenation (StO_2_), near-infrared perfusion index (NIR PI), and other parameters, did not indicate any disadvantage in perfusion associated with umbilicopexy. Interestingly, intraoperative StO_2_ values were slightly higher in the umbilicopexy group, although this difference was not observed postoperatively. This finding could indicate a transient effect influenced by surgical technique or other intraoperative factors.

Postoperative perfusion changes, as demonstrated by a general decline in NIR PI and StO_2_ from intraoperative to postoperative day 2, highlight the dynamic nature of tissue perfusion during the immediate recovery period.^5^ These changes likely reflect physiological responses to surgery, emphasizing the utility of HSI as a tool for monitoring tissue perfusion in real time.^6^

Our results align with existing literature underscoring the utility of HSI in assessing tissue viability and perfusion in various surgical contexts.^7^ However, the technology is not without limitations. HSI provides a superficial analysis of tissue perfusion with limited penetration depth and may be influenced by external factors such as patients skin tone, ambient light, temperature, and tissue surface conditions.^8^ Additionally, our study was conducted in a single-center setting with a relatively small sample size, which may limit the generalizability of our findings. Of note, the control group had more higher degree obese patients in absolute numbers but the subgroups were too small for a sound analysis in order to detect true differences. There are several potential confounding factors that have not been assessed by this study and that may influence umbilical perfusion and oxygenation, including pedicle length and diameter, the amount of surrounding tissue, potential pedicle kinking in massive weight loss patients, and the thickness of the abdominal flap, which may affect tension on the umbilicus when fixed to the fascia. While these variables were not explicitly analyzed, they represent important considerations that warrant further investigation. However, this is exactly where HSI comes into play as it directly measures perfusion and not a parameter that may or may not constitute a risk factor.

Future studies with larger cohorts are necessary to validate these preliminary results and further investigate the long-term implications of umbilicopexy on surgical outcomes.

Because of these limitations for now no conclusion about the safety in regards of local complication of umbilicopexy in abdominoplasty, addressing a critical concern regarding the potential compromise of umbilical perfusion, is possible. Previous literature has highlighted the vulnerability of the umbilicus to ischemic complications, for instance in post-bariatric patients with elongated umbilical stalks and altered vascular anatomy.^9^ Techniques such as umbilicopexy have often been debated, with limited evidence to support their safety or efficacy in preserving perfusion.^1^ Compared to other studies utilizing non-invasive imaging modalities to assess perfusion, our use of hyperspectral imaging (HSI) provided real-time, quantitative insights, adding a novel dimension to the assessment of tissue viability in this context.^10^

The absence of adverse effects on perfusion and wound healing suggests that umbilicopexy can be safely performed to achieve desired aesthetic outcomes in abdominoplasty, however due to the small sample size and several uncontrolled factors, a definitive conclusion regarding the safety of umbilicopexy realating local complications is not possible. By providing objective data on tissue perfusion, HSI offers a valuable adjunct for intraoperative and postoperative monitoring, potentially enhancing patient outcomes and reducing the risk of complications in the future. Additionally using HSI for intraoperative decision making wether or not to perform a umbilicopexy is intended for future use.

## Conclusion

This study suggests that umbilical fixation to the fascia (umbilicopexy) does not negatively impact umbilical perfusion or wound healing in the context of abdominoplasty. Using hyperspectral imaging (HSI), we found no significant differences in tissue perfusion indices or clinical outcomes between the umbilicopexy and control group. These findings implythat umbilicopexy can be safely incorporated into surgical practice to enhance aesthetic results without compromising umbilical vascularity. While our results are promising, further research with larger cohorts, additional confounding factors and long-term follow-up is warranted to confirm these findings and explore the broader implications of HSI in surgical decision-making and postoperative monitoring.

## Funding

No funding was received for the measurements and/or preparation of this manuscript.

## Availability of data

All raw data can be provided by the corresponding author upon request.

## Ethics approval

The protocol of this prospective diagnostic study was approved by the local ethics committee (426/19-ek).

## Consent to participate and publication

All participants gave informed consent to the use of their data and images for analysis and publication.

## Declaration of generative AI usage

During the preparation of this work the author(s) used ChatGPT in order to optimize language. After using this tool/service, the author(s) reviewed and edited the content as needed and take(s) full responsibility for the content of the publication.

## Author contributions

MK participated in designing the study, data acquisition and data analysis and drafted part of the manuscript. HK participated in data acquisition and data analysis and revised the manuscript. LK, RN and TS participated in designing the study, patient recruiting and data acquisition and revised the manuscript. GO had the idea for the study, participated in designing the study, patient recruiting, data acquisition and data analysis and drafted part of the manuscript.

## Declaration of competing interest

The authors have no conflicts of interest to declare.
